# 4-Amino­pyridinium *cis*-2-carb­oxy­cyclo­hexane-1-carboxyl­ate

**DOI:** 10.1107/S1600536811039547

**Published:** 2011-09-30

**Authors:** Graham Smith, Urs D. Wermuth

**Affiliations:** a Faculty of Science and Technology, Queensland University of Technology, GPO Box 2434, Brisbane, Queensland 4001, Australia

## Abstract

In the structure of the title molecular salt, C_5_H_7_N_2_^+^·C_8_H_11_O_4_^−^, the *cis* monoanions associate through short O—H⋯O hydrogen bonds in the carb­oxy­lic acid groups [graph set *C*(7)], forming zigzag chains which extend along the *c* axis. These are inter­linked through pyridinium and amine N—H⋯O hydrogen bonds, giving a three-dimensional network structure.

## Related literature

For the structure of racemic *cis*-cyclo­hexane-1,2-dicarb­oxy­lic acid, see: Benedetti *et al.* (1970 ▶). For the structure of the racemic ammonium and 2-amino­pyridinium salts of *cis*-2-carb­oxy­cyclo­hexane-1-carboxyl­ate, see: Smith & Wermuth (2011*a* ▶,*b* ▶). For graph-set analysis, see Etter *et al.* (1990 ▶). 
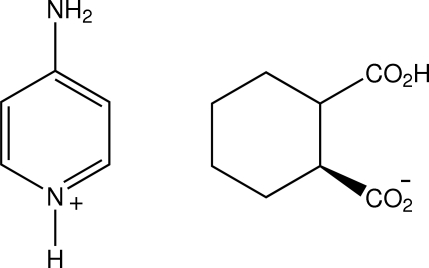


## Experimental

### 

#### Crystal data

C_5_H_7_N_2_^+^·C_8_H_11_O_4_^−^
*M* *_r_* = 266.29
Orthorhombic, 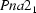

*a* = 12.1359 (3) Å
*b* = 9.8351 (3) Å
*c* = 11.1850 (3) Å
*V* = 1335.02 (6) Å^3^
*Z* = 4
Mo *K*α radiation
μ = 0.10 mm^−1^
*T* = 200 K
0.30 × 0.25 × 0.20 mm

#### Data collection

Oxford Diffraction Gemini-S CCD-detector diffractometer
Absorption correction: multi-scan (*CrysAlis PRO*; Oxford Diffraction, 2010 ▶) *T*_min_ = 0.948, *T*_max_ = 0.990
9670 measured reflections
1709 independent reflections
1448 reflections with *I* > 2σ(*I*)
*R*_int_ = 0.029

#### Refinement

*R*[*F* ^2^ > 2σ(*F* ^2^)] = 0.028
*wR*(*F* ^2^) = 0.060
*S* = 0.99
1709 reflections
188 parameters
1 restraint
H atoms treated by a mixture of independent and constrained refinement
Δρ_max_ = 0.15 e Å^−3^
Δρ_min_ = −0.16 e Å^−3^

### 

Data collection: *CrysAlis PRO* (Oxford Diffraction, 2010 ▶); cell refinement: *CrysAlis PRO*; data reduction: *CrysAlis PRO*; program(s) used to solve structure: *SIR92* (Altomare *et al.*, 1994 ▶); program(s) used to refine structure: *SHELXL97* (Sheldrick, 2008 ▶) within *WinGX* (Farrugia, 1999 ▶); molecular graphics: *PLATON* (Spek, 2009 ▶); software used to prepare material for publication: *PLATON*.

## Supplementary Material

Crystal structure: contains datablock(s) global, I. DOI: 10.1107/S1600536811039547/fj2453sup1.cif

Structure factors: contains datablock(s) I. DOI: 10.1107/S1600536811039547/fj2453Isup2.hkl

Supplementary material file. DOI: 10.1107/S1600536811039547/fj2453Isup3.cml

Additional supplementary materials:  crystallographic information; 3D view; checkCIF report

## Figures and Tables

**Table 1:** Hydrogen-bond geometry (Å, °)

*D*—H⋯*A*	*D*—H	H⋯*A*	*D*⋯*A*	*D*—H⋯*A*
N1*A*—H1*A*⋯O12^i^	0.88 (2)	1.91 (2)	2.795 (2)	180 (3)
N41*A*—H41*A*⋯O12^ii^	0.86 (2)	2.14 (2)	2.989 (2)	168 (2)
N41*A*—H42*A*⋯O22	0.91 (2)	2.13 (2)	2.974 (2)	152.6 (18)
O21—H21⋯O11^iii^	0.95 (3)	1.59 (3)	2.5302 (17)	170 (3)

Symmetry codes: (i) 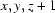; (ii) 
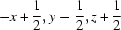
; (iii) 
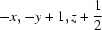
.

**Fig. 1.**
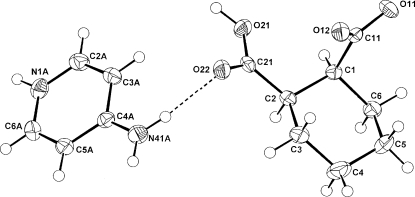
Molecular configuration and atom naming scheme for the cation the anion species in the title salt. Inter-species hydrogen bonds are shown as dashed lines and displacement ellipsoids are drawn at the 50% probability level.

**Fig. 2.**
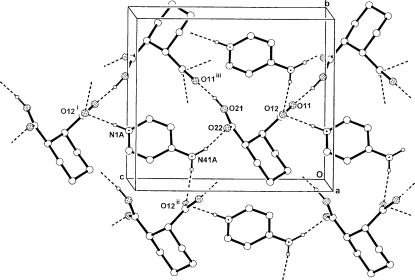
A perspective view of the unit cell showing the hydrogen-bonded zigzag C(7) cis-CHDC monoanion chains and their inter-linking cations, with hydrogen bonds shown as dashed lines. Non-associative H atoms are omitted. For symmetry codes, see Table 1.

**Fig. 3.**
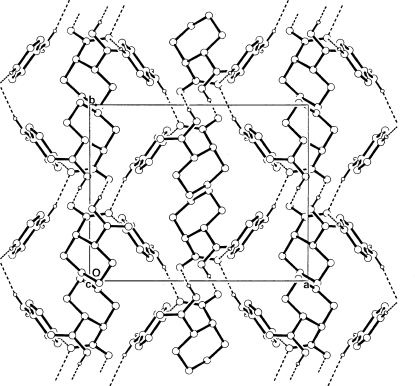
A view of the hydrogen-bonded structure looking down the c axis.

## supplementary crystallographic information

### Comment

The structures of Lewis base salts of *cis*-cyclohexane-1,2-dicarboxylic acid (*cis*-CHDC) are rare in the crystallographic literature and like the parent *cis*-acid (Benedetti *et al.*, 1970), exist only in the unresolved racemic form. We have reported the structures of the 1:1 ammonium salt (Smith & Wermuth, 2011*a*) and the 1:1 2-aminopyridinium salt (Smith & Wermuth, 2011*b*) and in our parallel 1:1 stoichiometric reaction of *cis*-CHDC anhydride with 4-aminopyridine in 50% ethanol–water solution we also obtained minor crystals of the title compound, *cis*-C_5_H_7_N_2_^+^ C_8_H_11_O_4_^-^ (Fig. 1) and the structure is reported here.

In the structure of the title compound, the monoanions associate through strong carboxylic acid–carboxyl O—H···O hydrogen bonds (Table 1) giving zigzag chains [graph set *C*(7) (Etter *et al.*, 1990)] which extend along *c* (Fig. 2). The cations provide links between these chains through both pyridinium and amine *N*—*H*···O_carboxyl_ hydrogen bonds, resulting in a three-dimensional structure (Figs. 2,3).

### Experimental

The title compound was synthesized by heating a solution of 1 mmol of cyclohexane-1,2-dicarboxylic anhydride and 1 mmol of 4-aminopyridine in 50 ml of 1:1 ethanol–water under reflux for 10 min. After concentration to 30 ml the solution was allowed to evaporate at room temperature, giving finally a residual viscous oil in which minor well formed colourless crystals of the title compound were found.

### Refinement

Hydrogen atoms involved in hydrogen-bonding interactions were located by difference methods and their positional and isotropic displacement parameters were refined. Other H-atoms were included in the refinement at calculated positions [C–H = 0.93–0.98 Å] and with *U*_iso_(H) = 1.2*U*_eq_(C), using a riding-model approximation. In the absence of a suitable heavy atom in the structure, the Friedel pairs (1332) were merged for the final cycles of the refinement. In the structure reported here, the *cis*-CHDC anion has the (1*S*,2*R*) configuration.

### Crystal data

**Table:**

C_5_H_7_N_2_^+^·C_8_H_11_O_4_^−^	*F*(000) = 568
*M**_r_* = 266.29	*D*_x_ = 1.325 Mg m^−^^3^
Orthorhombic, *P**n**a*2_1_	Mo *K*α radiation, λ = 0.71073 Å
Hall symbol: P 2c -2n	Cell parameters from 4840 reflections
*a* = 12.1359 (3) Å	θ = 3.2–28.7°
*b* = 9.8351 (3) Å	µ = 0.10 mm^−^^1^
*c* = 11.1850 (3) Å	*T* = 200 K
*V* = 1335.02 (6) Å^3^	Block, colourless
*Z* = 4	0.30 × 0.25 × 0.20 mm

### Data collection

**Table:**

Oxford Diffraction Gemini-S CCD-detector diffractometer	1709 independent reflections
Radiation source: Enhance (Mo) X-ray source	1448 reflections with *I* > 2σ(*I*)
graphite	*R*_int_ = 0.029
Detector resolution: 16.077 pixels mm^-1^	θ_max_ = 28.8°, θ_min_ = 3.2°
ω scans	*h* = −16→15
Absorption correction: multi-scan (*CrysAlis PRO*; Oxford Diffraction, 2010)	*k* = −12→13
*T*_min_ = 0.948, *T*_max_ = 0.990	*l* = −13→15
9670 measured reflections	

### Refinement

**Table:**

Refinement on *F*^2^	Primary atom site location: structure-invariant direct methods
Least-squares matrix: full	Secondary atom site location: difference Fourier map
*R*[*F*^2^ > 2σ(*F*^2^)] = 0.028	Hydrogen site location: inferred from neighbouring sites
*wR*(*F*^2^) = 0.060	H atoms treated by a mixture of independent and constrained refinement
*S* = 0.99	*w* = 1/[σ^2^(*F*_o_^2^) + (0.0357*P*)^2^] where *P* = (*F*_o_^2^ + 2*F*_c_^2^)/3
1709 reflections	(Δ/σ)_max_ < 0.001
188 parameters	Δρ_max_ = 0.15 e Å^−^^3^
1 restraint	Δρ_min_ = −0.16 e Å^−^^3^

### Special details

**Table:**

Geometry. Bond distances, angles *etc*. have been calculated using the rounded fractional coordinates. All su's are estimated from the variances of the (full) variance-covariance matrix. The cell e.s.d.'s are taken into account in the estimation of distances, angles and torsion angles
Refinement. Refinement of *F*^2^ against ALL reflections. The weighted *R*-factor *wR* and goodness of fit *S* are based on *F*^2^, conventional *R*-factors *R* are based on *F*, with *F* set to zero for negative *F*^2^. The threshold expression of *F*^2^ > σ(*F*^2^) is used only for calculating *R*-factors(gt) *etc*. and is not relevant to the choice of reflections for refinement. *R*-factors based on *F*^2^ are statistically about twice as large as those based on *F*, and *R*- factors based on ALL data will be even larger.

### Fractional atomic coordinates and isotropic or equivalent isotropic displacement parameters (Å^2^)

**Table:**

	*x*	*y*	*z*	*U*_iso_*/*U*_eq_	
O11	−0.08623 (9)	0.42384 (12)	0.16082 (11)	0.0293 (4)	
O12	0.08627 (8)	0.38250 (12)	0.21757 (10)	0.0262 (3)	
O21	0.01171 (10)	0.40723 (12)	0.51058 (12)	0.0285 (3)	
O22	0.17703 (9)	0.30714 (12)	0.50187 (12)	0.0296 (3)	
C1	−0.06239 (12)	0.25883 (16)	0.31330 (14)	0.0202 (4)	
C2	0.02258 (13)	0.20096 (16)	0.40246 (15)	0.0223 (5)	
C3	0.10393 (14)	0.10098 (18)	0.34520 (18)	0.0311 (5)	
C4	0.04378 (15)	−0.01169 (19)	0.2767 (2)	0.0410 (6)	
C5	−0.03704 (15)	0.04676 (19)	0.18597 (18)	0.0340 (6)	
C6	−0.11879 (14)	0.14099 (17)	0.24603 (17)	0.0272 (5)	
C11	−0.01574 (12)	0.36284 (16)	0.22481 (14)	0.0200 (5)	
C21	0.07930 (13)	0.30994 (17)	0.47466 (14)	0.0222 (5)	
N1A	0.16603 (12)	0.28917 (15)	0.99803 (15)	0.0301 (4)	
N41A	0.30073 (13)	0.14997 (18)	0.68510 (15)	0.0320 (5)	
C2A	0.14635 (14)	0.35553 (19)	0.89470 (16)	0.0306 (5)	
C3A	0.18908 (13)	0.31222 (17)	0.78984 (16)	0.0285 (5)	
C4A	0.25659 (12)	0.19462 (16)	0.78670 (15)	0.0236 (5)	
C5A	0.27516 (14)	0.12848 (18)	0.89698 (16)	0.0289 (5)	
C6A	0.22940 (14)	0.17671 (18)	0.99826 (17)	0.0309 (5)	
H1	−0.11940	0.30510	0.36020	0.0240*	
H2	−0.02010	0.14740	0.46010	0.0270*	
H21	0.046 (2)	0.473 (3)	0.561 (3)	0.069 (8)*	
H31B	0.15200	0.14990	0.29080	0.0370*	
H32B	0.14940	0.06050	0.40700	0.0370*	
H41B	0.09730	−0.06800	0.23550	0.0490*	
H42B	0.00420	−0.06880	0.33280	0.0490*	
H51B	0.00320	0.09640	0.12510	0.0410*	
H52B	−0.07640	−0.02690	0.14720	0.0410*	
H61B	−0.16300	0.08910	0.30200	0.0330*	
H62B	−0.16790	0.17830	0.18600	0.0330*	
H1A	0.1408 (18)	0.319 (2)	1.0674 (19)	0.038 (6)*	
H2A	0.10230	0.43290	0.89560	0.0370*	
H3A	0.17420	0.35960	0.71970	0.0340*	
H5A	0.31920	0.05120	0.89970	0.0350*	
H6A	0.24190	0.13130	1.06990	0.0370*	
H41A	0.3409 (18)	0.078 (2)	0.687 (2)	0.047 (6)*	
H42A	0.2841 (16)	0.193 (2)	0.615 (2)	0.038 (6)*	

### Atomic displacement parameters (Å^2^)

**Table:**

	*U*^11^	*U*^22^	*U*^33^	*U*^12^	*U*^13^	*U*^23^
O11	0.0297 (6)	0.0270 (6)	0.0312 (7)	0.0010 (5)	−0.0051 (5)	0.0106 (6)
O12	0.0244 (6)	0.0291 (6)	0.0251 (6)	−0.0047 (5)	0.0004 (5)	0.0028 (5)
O21	0.0321 (6)	0.0263 (6)	0.0272 (6)	0.0042 (5)	−0.0021 (6)	−0.0066 (6)
O22	0.0304 (6)	0.0316 (6)	0.0269 (6)	0.0036 (5)	−0.0062 (5)	−0.0019 (6)
C1	0.0205 (7)	0.0188 (8)	0.0212 (8)	0.0009 (6)	0.0009 (6)	−0.0003 (7)
C2	0.0267 (8)	0.0193 (8)	0.0209 (8)	−0.0009 (7)	0.0007 (7)	0.0046 (7)
C3	0.0325 (9)	0.0253 (9)	0.0354 (10)	0.0084 (8)	−0.0064 (8)	−0.0016 (8)
C4	0.0439 (10)	0.0247 (9)	0.0543 (13)	0.0080 (8)	−0.0046 (10)	−0.0118 (9)
C5	0.0387 (10)	0.0270 (9)	0.0363 (11)	−0.0064 (8)	−0.0040 (9)	−0.0106 (8)
C6	0.0269 (8)	0.0226 (8)	0.0322 (10)	−0.0068 (7)	−0.0053 (7)	0.0039 (8)
C11	0.0254 (8)	0.0166 (8)	0.0179 (8)	−0.0006 (6)	−0.0006 (6)	−0.0031 (6)
C21	0.0287 (8)	0.0222 (8)	0.0157 (8)	0.0017 (7)	−0.0004 (6)	0.0051 (7)
N1A	0.0318 (7)	0.0344 (8)	0.0240 (8)	0.0016 (6)	0.0021 (7)	−0.0067 (8)
N41A	0.0366 (8)	0.0293 (9)	0.0302 (9)	0.0098 (7)	0.0024 (7)	−0.0044 (7)
C2A	0.0324 (9)	0.0258 (9)	0.0335 (10)	0.0077 (8)	0.0012 (8)	−0.0007 (9)
C3A	0.0334 (9)	0.0241 (9)	0.0281 (9)	0.0057 (7)	−0.0019 (8)	0.0023 (8)
C4A	0.0225 (7)	0.0209 (8)	0.0275 (9)	−0.0010 (6)	−0.0021 (7)	−0.0032 (8)
C5A	0.0305 (9)	0.0233 (9)	0.0329 (10)	0.0054 (7)	−0.0085 (8)	−0.0012 (8)
C6A	0.0334 (9)	0.0328 (9)	0.0264 (9)	−0.0001 (8)	−0.0080 (8)	0.0013 (9)

### Geometric parameters (Å, °)

**Table:**

O11—C11	1.2665 (19)	C1—H1	0.9800
O12—C11	1.2556 (18)	C2—H2	0.9800
O21—C21	1.323 (2)	C3—H32B	0.9700
O22—C21	1.2248 (19)	C3—H31B	0.9700
O21—H21	0.95 (3)	C4—H42B	0.9700
N1A—C6A	1.347 (2)	C4—H41B	0.9700
N1A—C2A	1.349 (2)	C5—H51B	0.9700
N41A—C4A	1.331 (2)	C5—H52B	0.9700
N1A—H1A	0.88 (2)	C6—H61B	0.9700
N41A—H41A	0.86 (2)	C6—H62B	0.9700
N41A—H42A	0.91 (2)	C2A—C3A	1.351 (2)
C1—C2	1.543 (2)	C3A—C4A	1.418 (2)
C1—C11	1.532 (2)	C4A—C5A	1.413 (2)
C1—C6	1.542 (2)	C5A—C6A	1.348 (3)
C2—C3	1.534 (2)	C2A—H2A	0.9300
C2—C21	1.508 (2)	C3A—H3A	0.9300
C3—C4	1.532 (3)	C5A—H5A	0.9300
C4—C5	1.524 (3)	C6A—H6A	0.9300
C5—C6	1.515 (3)		
			
C21—O21—H21	113.6 (15)	C2—C3—H31B	109.00
C2A—N1A—C6A	120.01 (16)	C2—C3—H32B	109.00
C6A—N1A—H1A	117.9 (13)	C5—C4—H41B	109.00
C2A—N1A—H1A	122.0 (13)	C5—C4—H42B	109.00
H41A—N41A—H42A	122 (2)	C3—C4—H42B	109.00
C4A—N41A—H41A	118.6 (15)	C3—C4—H41B	109.00
C4A—N41A—H42A	119.4 (13)	H41B—C4—H42B	108.00
C2—C1—C6	109.55 (13)	C6—C5—H51B	109.00
C2—C1—C11	114.64 (12)	C4—C5—H52B	109.00
C6—C1—C11	110.53 (13)	C6—C5—H52B	109.00
C1—C2—C21	112.88 (13)	H51B—C5—H52B	108.00
C1—C2—C3	113.38 (14)	C4—C5—H51B	109.00
C3—C2—C21	112.67 (13)	C5—C6—H62B	109.00
C2—C3—C4	111.46 (14)	H61B—C6—H62B	108.00
C3—C4—C5	111.52 (15)	C1—C6—H61B	109.00
C4—C5—C6	110.92 (16)	C1—C6—H62B	109.00
C1—C6—C5	112.68 (14)	C5—C6—H61B	109.00
O11—C11—C1	115.58 (13)	N1A—C2A—C3A	121.56 (17)
O11—C11—O12	123.82 (14)	C2A—C3A—C4A	120.03 (16)
O12—C11—C1	120.59 (13)	N41A—C4A—C3A	121.53 (16)
O21—C21—C2	113.18 (13)	C3A—C4A—C5A	116.50 (15)
O22—C21—C2	123.99 (15)	N41A—C4A—C5A	121.97 (15)
O21—C21—O22	122.77 (15)	C4A—C5A—C6A	120.41 (16)
C6—C1—H1	107.00	N1A—C6A—C5A	121.50 (17)
C2—C1—H1	107.00	N1A—C2A—H2A	119.00
C11—C1—H1	107.00	C3A—C2A—H2A	119.00
C1—C2—H2	106.00	C2A—C3A—H3A	120.00
C3—C2—H2	106.00	C4A—C3A—H3A	120.00
C21—C2—H2	106.00	C4A—C5A—H5A	120.00
C4—C3—H32B	109.00	C6A—C5A—H5A	120.00
H31B—C3—H32B	108.00	N1A—C6A—H6A	119.00
C4—C3—H31B	109.00	C5A—C6A—H6A	119.00
			
C6A—N1A—C2A—C3A	−0.2 (3)	C3—C2—C21—O21	171.15 (14)
C2A—N1A—C6A—C5A	0.6 (3)	C3—C2—C21—O22	−11.6 (2)
C6—C1—C2—C3	51.98 (17)	C1—C2—C21—O21	41.13 (19)
C11—C1—C2—C21	56.73 (18)	C1—C2—C21—O22	−141.58 (16)
C6—C1—C2—C21	−178.36 (13)	C2—C3—C4—C5	53.7 (2)
C11—C1—C2—C3	−72.93 (17)	C3—C4—C5—C6	−56.2 (2)
C2—C1—C11—O11	−171.66 (14)	C4—C5—C6—C1	57.4 (2)
C2—C1—C11—O12	9.2 (2)	N1A—C2A—C3A—C4A	−0.2 (3)
C6—C1—C11—O11	63.95 (18)	C2A—C3A—C4A—N41A	−179.40 (16)
C6—C1—C11—O12	−115.22 (16)	C2A—C3A—C4A—C5A	0.0 (2)
C11—C1—C6—C5	72.82 (18)	N41A—C4A—C5A—C6A	179.81 (17)
C2—C1—C6—C5	−54.43 (19)	C3A—C4A—C5A—C6A	0.4 (2)
C21—C2—C3—C4	177.72 (15)	C4A—C5A—C6A—N1A	−0.7 (3)
C1—C2—C3—C4	−52.5 (2)		

### Hydrogen-bond geometry (Å, °)

**Table:**

*D*—H···*A*	*D*—H	H···*A*	*D*···*A*	*D*—H···*A*
N1A—H1A···O12^i^	0.88 (2)	1.91 (2)	2.795 (2)	180 (3)
N41A—H41A···O12^ii^	0.86 (2)	2.14 (2)	2.989 (2)	168 (2)
N41A—H42A···O22	0.91 (2)	2.13 (2)	2.974 (2)	152.6 (18)
O21—H21···O11^iii^	0.95 (3)	1.59 (3)	2.5302 (17)	170 (3)
C2A—H2A···O21^iii^	0.93	2.46	3.287 (2)	149
C3A—H3A···O22	0.93	2.49	3.225 (2)	136
C3A—H3A···O11^iii^	0.93	2.47	3.222 (2)	138
C6A—H6A···O11^iv^	0.93	2.38	3.048 (2)	128
C3—H31B···O12	0.97	2.56	3.123 (2)	117

Symmetry codes: (i) *x*, *y*, *z*+1; (ii) −*x*+1/2, *y*−1/2, *z*+1/2; (iii) −*x*, −*y*+1, *z*+1/2; (iv) *x*+1/2, −*y*+1/2, *z*+1.
